# Predictive Value of Point-of-Care Proenkephalin for Worsening Renal Function and Mortality in Patients Presenting to Emergency Department with Acute Heart Failure

**DOI:** 10.3390/jcm14165730

**Published:** 2025-08-13

**Authors:** Dionysis Matsiras, Effie Polyzogopoulou, Ioannis Ventoulis, Vasiliki Bistola, Christos Verras, Ignatios Ikonomidis, John Parissis

**Affiliations:** 1 Department of Emergency Medicine, Attikon University Hospital, National and Kapodistrian University of Athens, Rimini 1, 12462 Athens, Greece; mats.dionysis@gmail.com (D.M.); effiepol@med.uoa.gr (E.P.); vasobistola@yahoo.com (V.B.); christos.verras@gmail.com (C.V.); 2 Department of Occupational Therapy, University of Western Macedonia, Keptse Area, 50200 Ptolemaida, Greece; iventoulis@uowm.gr; 3 2nd Department of Cardiology, Medical School, Attikon University Hospital, National and Kapodistrian University of Athens, 12462 Athens, Greece; ignoik@gmail.com

**Keywords:** proenkephalin, acute heart failure, point-of-care testing, worsening renal function, prognostic biomarker, emergency department

## Abstract

**Background:** Enkephalins are endogenous opioid peptides that modulate cardiovascular and renal function and are overexpressed in patients with acute heart failure (AHF). Although biologically active enkephalins lack a favorable biomarker profile, their stable surrogate proenkephalin 119–159 (PENK) appears to display prognostic value in AHF settings. The aim of the present study was to evaluate the role of point-of-care (POC) PENK in predicting mortality and worsening renal function (WRF) in patients presenting to the emergency department (ED) with AHF. **Methods:** In this single-center observational study, 107 patients presenting to the ED with AHF were prospectively enrolled. We measured PENK levels upon ED presentation with a commercially available POC immunoassay and investigated their association with WRF within 48 h and all-cause mortality during a 1-year follow-up. **Results:** The patients had a mean age of 72 ± 13 years, and 58% were men. Moreover, 62% had acutely decompensated chronic heart failure (HF), 24% had pulmonary edema, 9% had cardiogenic shock, and 5% had right HF. The median PENK levels were 111 [60–193] pmol/L. PENK was independently associated with WRF (adjusted OR, 95% CI: 15.4 [2.0–120.2]; *p* = 0.009), with levels of ≥90.5 pmol/L identified as the optimal cut-off for predicting WRF (AUC: 0.690; *p* < 0.001). PENK was also an independent predictor of short- and long-term all-cause mortality, with an optimal cut-off of ≥95.8 pmol/L (AUC for 30-day, 90-day, and 1-year mortality: 0.717, 0.723, and 0.724, respectively; all *p* < 0.001). **Conclusions:** In patients presenting to the ED with AHF, POC PENK may serve as an early prognostic marker of WRF and short- and long-term mortality.

## 1. Introduction

Heart failure (HF) constitutes a major cause of morbidity and mortality worldwide and is characterized by frequent hospitalizations and progressive deterioration, leading to substantial in-hospital and post-discharge fatality rates [1]. Moreover, cardiac dysfunction and the subsequent neurohormonal perturbations have a detrimental effect on kidney function, manifesting as cardiorenal syndrome. Worsening renal function (WRF) complicates the hospitalization of approximately 30% of patients admitted for acute heart failure (AHF) and further aggravates their prognosis, since it leads to increased length of stay, readmission rates, and mortality [2]. Conventional biomarkers of kidney function result in diagnostic delays in terms of identifying WRF [3] and in suboptimal patient stratification with regard to short-term prognosis. In order to improve the management of patients with AHF in the ED and optimize patient outcomes, novel biomarkers are being investigated for their ability to identify those patients who are at high risk of WRF or death [4].

Enkephalins are endogenous opioid peptides that exert various physiological effects, including the regulation of cardiovascular and renal function, by acting on specific G protein-coupled opioid receptors (ORs). Interestingly, during HF, the maladaptive adrenergic overstimulation is accompanied by an upregulation of both the enkephalin peptides and their corresponding ORs [5]. The association between AHF severity and enkephalin plasma levels renders enkephalins promising biomarkers [6], but their rapid in vitro degradation limits their applicability in routine clinical practice. In contrast, proenkephalin 119–159 (PENK) is a peptide generated as a by-product during the cleavage of the precursor hormone proenkephalin A, alongside the production of active enkephalin peptides. Although it is inactive per se, it has been regarded as a surrogate of the biologically active enkephalins due to the fact that it has a long half-life and is, thus, a more stable molecule in vitro [7]. Accordingly, the role of PENK has been studied in AHF populations, and increased levels of this biomarker have been found to be associated with higher rates of rehospitalization, WRF, and mortality [8]. Moreover, evidence supports the use of PENK as a real-time marker of renal function [9]. Taken altogether, these findings indicate that PENK may be used as an early risk-stratifying biomarker with regard to mortality and renal deterioration as soon as a patient presents to the emergency department (ED). Prior studies have employed assays with various turnaround times, precluding the use of this biomarker in the acute care setting. Recently, a novel point-of-care (POC) assay has become available; however, it has yet to be studied in the context of AHF.

The aim of the present study was to assess the clinical utility of PENK, measured with a POC assay at the bedside, in terms of predicting 30 d mortality, as well as long-term mortality, and WRF, when measured in patients with AHF promptly upon their presentation to the ED.

## 2. Materials and Methods

### 2.1. Study Design and Population

This study was conducted from May 2022 to November 2022. A total of 107 consecutive patients, presenting to the ED of a tertiary hospital (Attikon University Hospital, Athens, Greece), were recruited in this prospective observational study. The inclusion criteria included the diagnosis of AHF upon ED presentation and an age of >18 years. This study followed an all-comer design strategy, irrespective of the clinical phenotype of AHF, be it acute pulmonary edema, acutely decompensated chronic HF, cardiogenic shock, or isolated right HF. AHF diagnosis was established by cardiologists or emergency physicians, in accordance with the latest recommendations of the European Society of Cardiology [10]. Demographics and personal medical history were recorded by the attending physician. All enrolled patients underwent standard clinical and echocardiographic evaluation, while venous blood was drawn for routine laboratory and cardiac biomarker testing, namely, troponin and N-terminal pro B-type natriuretic peptide (NT-proBNP). Moreover, circulating PENK levels were measured in ethylenediaminetetraacetic acid (EDTA) whole-blood samples collected upon presentation. The 20 min long measurement was carried out at the bedside using a POC device.

Following initial management in the ED, patients were admitted either to the cardiology ward or the cardiac intensive care unit (ICU), depending on AHF severity and clinical judgement. PENK values were not used to guide clinical decision making or patient management at any point. Follow-up information regarding renal function, length of stay, in-hospital mortality, and 30-day mortality was obtained from the hospital-based patient registry and through phone interviews. Data regarding 90-day and 1-year mortality were also retrieved from the national electronic health record system. Patients who were under the age of 18 years, had coexisting sepsis, or a history of end-stage renal disease (ESRD) requiring hemodialysis, were excluded from this study.

The primary endpoint of this study was to determine the prognostic value of PENK in terms of predicting worsening renal function (WRF) and short-term (30 d) mortality. Secondary endpoints included associations between PENK and long-term mortality at 90 days and at 1 year.

This study was conducted according to the principles of the Declaration of Helsinki and was approved by the local Institutional Review Board (IRB) of Attikon University Hospital (IRB: ΕΒΔ281/5 May 2022). All participating patients or their next of kin provided written informed consent before enrolment in this study.

### 2.2. Routine Clinical and Laboratory Assessment

All study participants underwent routine clinical examinations, including assessment of vital signs, namely, systolic (SBP) and diastolic blood pressure (DBP), heart rate (HR), pulse oximetry (SpO_2_), respiratory rate (RR), and body temperature. A 12-lead electrocardiogram (ECG) was also performed. Laboratory testing included blood gas analysis, complete blood count (CBC), urea, creatinine, sodium, potassium, aspartate aminotransferase (AST), alanine aminotransferase (ALT), total bilirubin (TB), and routine cardiac and inflammatory biomarkers, namely, high-sensitivity cardiac troponin T (hs-cTnT), NT-proBNP, and C-reactive protein (CRP). The glomerular filtration rate (eGFR) was estimated using the 2021 Chronic Kidney Disease Epidemiology Collaboration (CKD-EPI) formula [11]. NT-proBNP was measured using an immunoassay method in a Preanalytics-COBAS 8000 (Roche Diagnostics, Basel, Switzerland) analyzer. All other laboratory exams were performed at the hospital’s central laboratory.

### 2.3. POC PENK Analysis

PENK analysis was performed using the IB10 Sphingotest^®^ penKid^®^ assay [12], which was designed for bedside testing by means of a commercially available automated POC device, the Nexus IB10 Analyzer (Nexus Dc Inc., San Diego, CA, USA). Results were obtained in 20 min. The POC PENK quantification assay requires 500 μL of EDTA whole-blood or plasma sample volume and is based on a chemiluminescence sandwich immunoassay reaction, previously described by Donato et al. [13]. The range of measured values for the IB10 POC assay is 50–500 pmol/L. The limit of blank (LoB), the limit of detection (LoD), and the limit of quantification (LoQ), or else the functional sensitivity at a 20% coefficient of variation (CV), of the POC assay are 21.3 pmol/L, 35.7 pmol/L, and 50 pmol/L respectively, as demonstrated by its analytical performance evaluation [12]. Compared with the reference method [13], POC measurements have been shown to be strongly correlated with it, exhibiting values of Pearson’s and Spearman’s correlation coefficients of r = 0.87 and rs = 0.86, respectively [12].

### 2.4. Echocardiography

Transthoracic echocardiography was performed with a Venue Go 1.75–3.5 MHz scanner (GE HealthCare Ultrasound, Chicago, IL, USA) with the use of harmonic imaging. Right-ventricular (RV) end-diastolic diameter, pulmonary artery systolic pressure (PASP), and inferior vena cava (IVC) measurements were performed as per current echocardiographic recommendations [14]. The left-ventricular ejection fraction (LVEF) was visually assessed. Patients with an LVEF ≤ 40%, 41–49%, or ≥50% were stratified as HF with a reduced ejection fraction (HFrEF), mildly reduced ejection fraction (HFmrEF), and preserved ejection fraction (HFpEF), respectively [10].

### 2.5. Statistical Analysis

Statistical analysis was performed using SPSS version 29.0 (SPSS, Inc., Chicago, IL, USA). Categorical variables are expressed as frequencies and percentages. Quantitative variables are reported as means with standard deviations (SDs) or medians with interquartile ranges (IQRs), depending on whether they were normally distributed or not, as tested by the Kolmogorov–Smirnov test. Owing to their highly skewed distribution, the values of laboratory variables were log-transformed for simple correlations and regression analyses. Logistic regression analysis and receiver operator characteristic (ROC) curves were used to examine the association between admission PENK and WRF within 48 h after hospital admission. WRF was defined as an absolute increase in creatinine levels of ≥0.3 mg/dl within 48 h after ED presentation or a relative increase of ≥50% compared with the baseline creatinine value within the same time frame. Univariate and multivariate Cox regression and Kaplan–Meier analyses were used to evaluate the prognostic value of PENK for all-cause mortality at 30 days, 90 days, and 1 year post-hospital admission. The Youden index was used to select the cut-offs of PENK for WRF and mortality prediction from ROC analyses. Post hoc power calculation showed that this study had 87.2% power to detect the observed difference in PENK levels between survivors and non-survivors at 30 days, at an alpha level of 0.05. A *p*-value of <0.05 was considered statistically significant.

## 3. Results

### 3.1. Patient Characteristics

One hundred and seven patients with AHF, without evidence of co-existing sepsis or a history of ESRD requiring hemodialysis, were prospectively enrolled upon ED presentation. Overall, 66 patients (62%) presented with acutely decompensated chronic HF, 26 (24%) with pulmonary edema, 10 (9%) with cardiogenic shock, and 5 (5%) with isolated RV failure. The mean (±SD) age of the total patient cohort was 72 ± 13 years, while 58% were males. Median PENK levels were 111 [60–193] pmol/L. The baseline characteristics of the total cohort and of the patient subgroups, categorized according to PENK tertiles, are shown in Table 1. Patients belonging to the third PENK tertile were older and presented with a more severe clinical picture and metabolic derangements than patients in the other tertiles. Patients in the third tertile presented more frequently with cardiogenic shock, had a higher need for invasive mechanical ventilation, and had lower SBP and DBP. Moreover, they had significantly higher serum urea, creatinine, and potassium levels, lower sodium concentrations, lower pH, and higher levels of cardiac biomarkers (NT-proBNP and hs-cTnT). No significant differences were observed between subgroups regarding the prevalence of comorbidities and focused echocardiography findings upon ED presentation (Table 1).

### 3.2. Association of PENK with Worsening Renal Function

**Figure 1:**
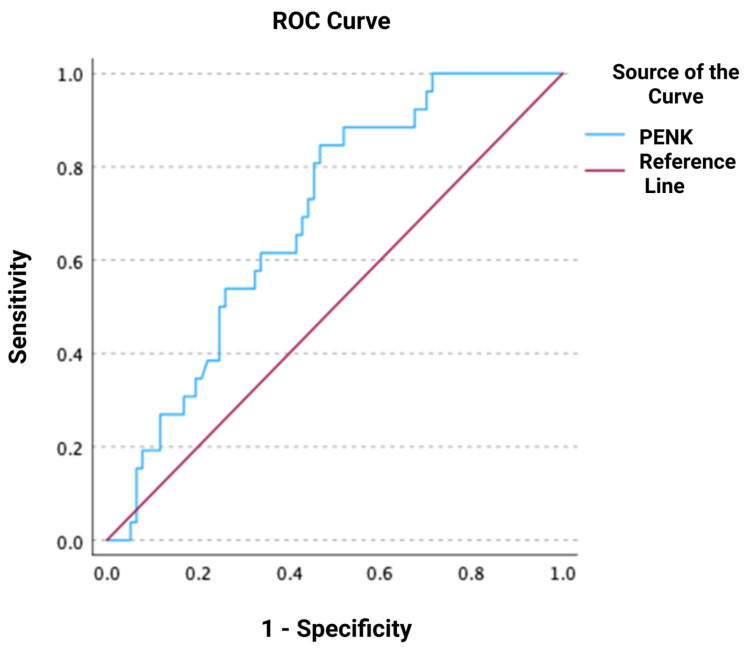
Receiver operating characteristic (ROC) curve of proenkephalin (PENK) for predicting worsening renal function.

**Figure 2:**
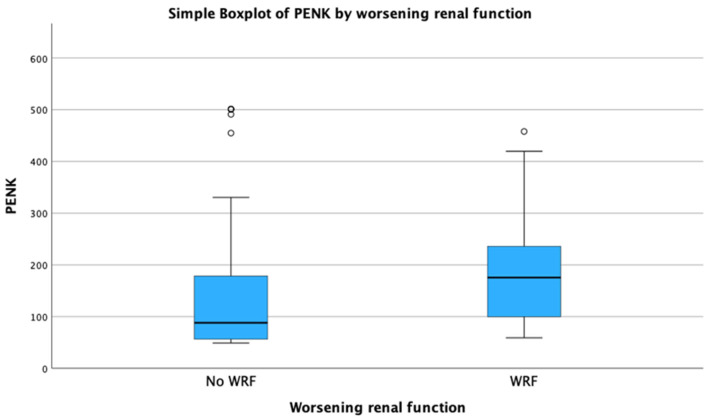
Distribution of proenkephalin (PENK) according to the presence or absence of worsening renal function.

In total, 26 out of 103 (25%) patients with available repeated creatinine measurements within 48 h after admission developed WRF. In the univariate logistic regression, logPENK was significantly associated with WRF, with an odds ratio (OR) and 95% confidence interval (CI) of 7.8 [1.7–36.1] (*p* = 0.009). This association remained significant after adjusting for other predictors of WRF, including SBP, eGFR, and logNT-proBNP (adjusted OR, 95% CI: 15.4 [2.0–120.2]; *p* = 0.009; chi-square = 9.509). During the ROC analysis, it was shown that PENK could predict WRF, with an AUC of 0.690 [0.584–0.795] (*p* < 0.001) (Figure 1). The optimal cut-off value of PENK for predicting WRF was determined at 90.5 pmol/L, yielding a sensitivity of 85%, specificity of 53%, negative predictive value (NPV) of 91%, positive predictive value (PPV) of 38%, positive likelihood ratio (LR^+^) of 1.81, and negative likelihood ratio (LR^-^) of 0.28. The distribution of PENK levels, in both patients who developed WRF and in those who did not, is shown in Figure 2.

### 3.3. Association of PENK with Mortality

**Figure 3:**
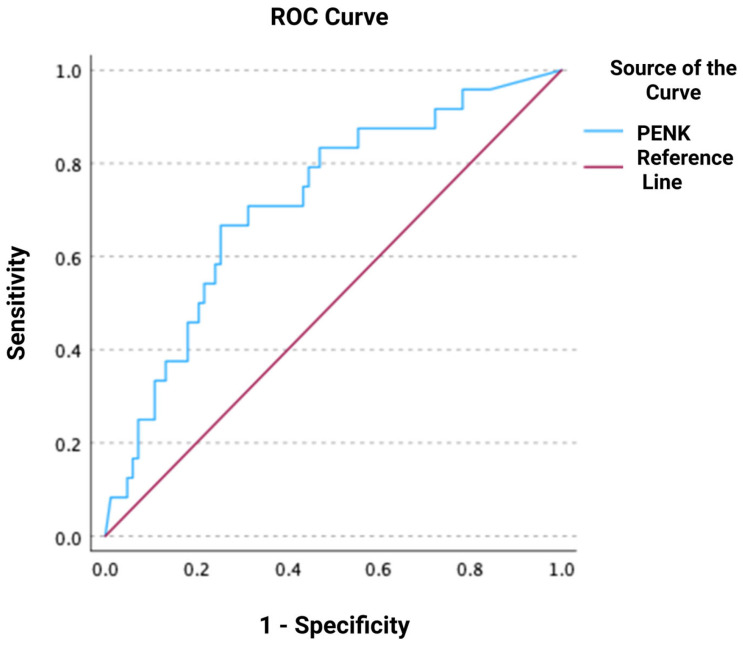
Receiver operating characteristic (ROC) curve of proenkephalin (PENK) for predicting all-cause mortality at 30 days.

**Figure 4:**
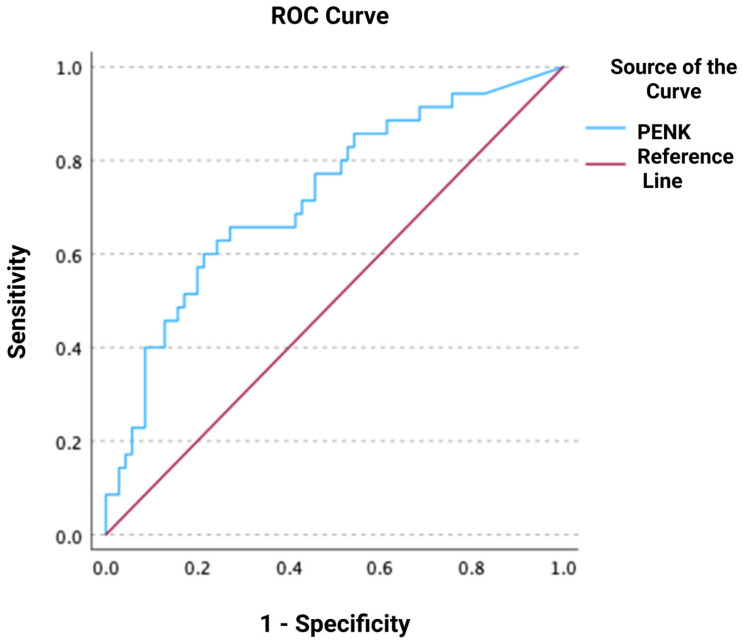
Receiver operating characteristic (ROC) curve of proenkephalin (PENK) for predicting all-cause mortality at 90 days.

**Figure 5:**
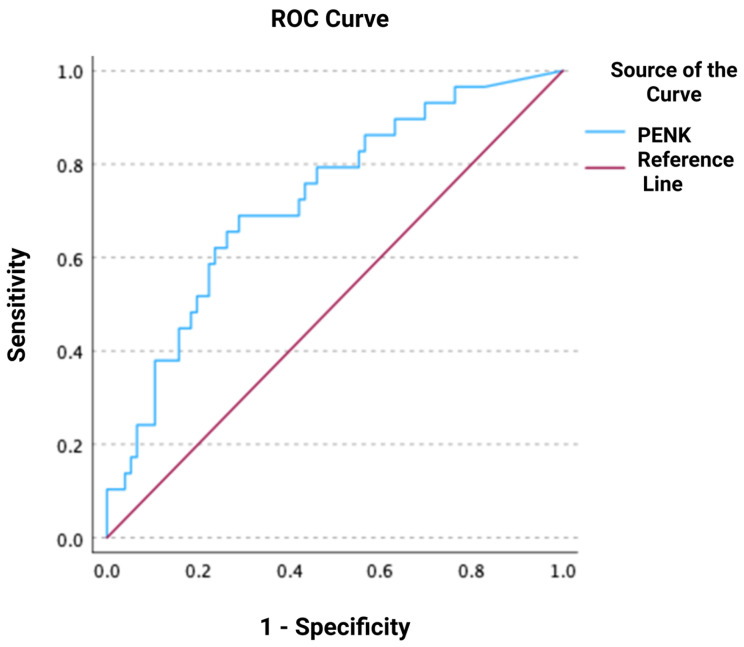
Receiver operating characteristic (ROC) curve of proenkephalin (PENK) for predicting all-cause mortality at 1 year.

**Figure 6:**
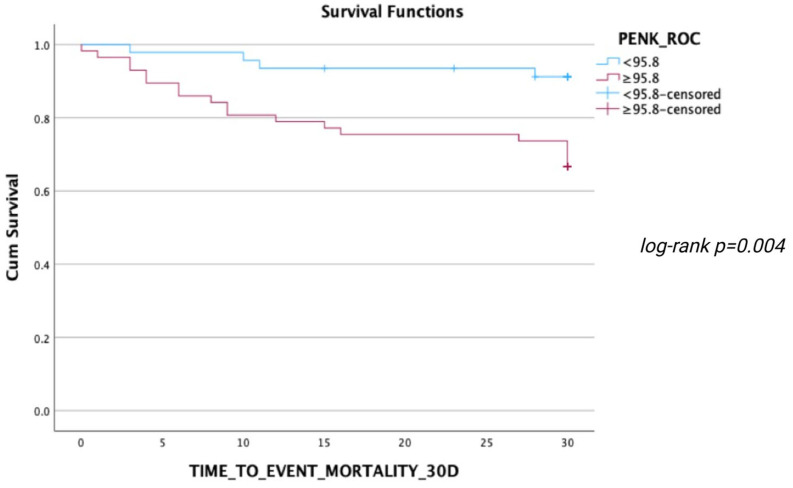
Kaplan–Meier 30-day survival curves for patients stratified by PENK levels (< or ≥95.8 pmol/L).

**Figure 7:**
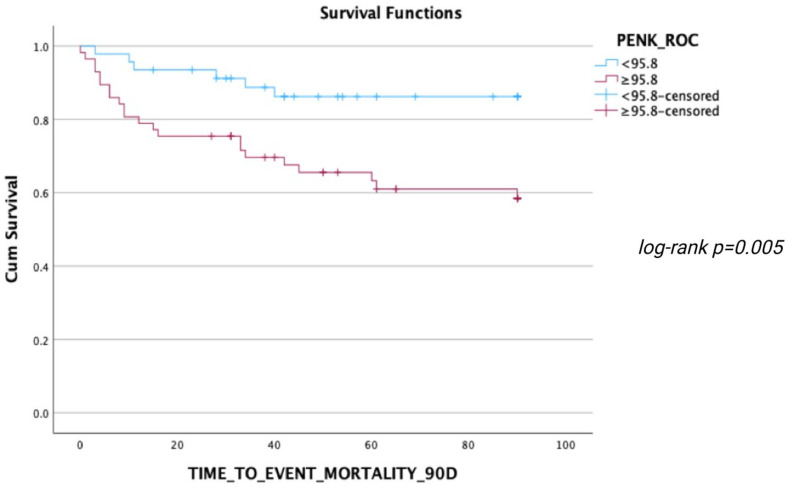
Kaplan–Meier 90-day survival curves for patients stratified by PENK levels (< or ≥95.8 pmol/L).

**Figure 8:**
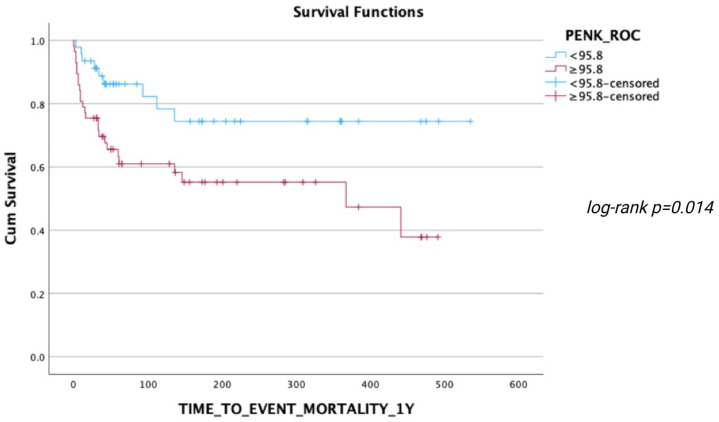
Kaplan–Meier 1-year survival curves for patients stratified by PENK levels (< or ≥95.8 pmol/L).

During the follow-up, 24 deaths occurred within 30 days, 29 deaths within 90 days, and 35 deaths within 1 year after ED presentation. ROC analyses regarding the predictive value of PENK for 30-day, 90-day, and 1-year mortality resulted in AUCs of 0.717 [0.602–0.832], 0.723 [0.616–0.831], and 0.724 [0.619–0.828], respectively (all *p* < 0.001) (Figure 3, Figure 4 and Figure 5). The optimal PENK cut-off value for predicting mortality at all time points was shown to be 95.8 pmol/L. With regard to the primary endpoint of 30 d mortality, the PENK cut-off of 95.8 pmol/L yielded 83% sensitivity, 53% specificity, 92% NPV, 33% PPV, 1.77 LR^+^, and 0.32 LR^-^. Regarding the secondary endpoints of 90d and 1-year mortality, the sensitivity, specificity, NPV, PPV, LR^+^, and LR^−^ were 79%, 54%, 87%, 39%, 1.72, and 0.39 for 90d mortality, while the corresponding values for 1-year mortality were 74%, 54%, 81%, 44%, 1.61, and 0.48, respectively. Figure 6, Figure 7 and Figure 8 depict Kaplan–Meier curves for all-cause mortality at 30 days, 90 days, and 1 year, when stratifying patients based on the PENK cut-off. In the univariate Cox regression, logPENK was significantly associated with 30-day, 90-day, and 1-year mortality (30 days: HR = 7.9 [2.2–29.3], *p* = 0.002; 90 days: HR = 8.0 [2.4–26.2], *p* < 0.001; 1 year: HR = 7.1 [2.4–20.9], *p* < 0.001). In the multivariable Cox regression, logPENK remained an independent predictor of mortality at all time points after adjustment for age, logNT-proBNP, and eGFR (30 days: adjusted HR = 6.5 [0.99–42.8], *p* = 0.051, chi-square = 12.413; 90 days: adjusted HR = 7.3 [1.5–35.0], *p* = 0.013, chi-square = 13.416; 1 year: adjusted HR = 7.2 [1.7–30.8], *p* = 0.008, chi-square = 17.761).

## 4. Discussion

In this prospective observational single-center study, we demonstrated that, in patients presenting to the ED with AHF, elevated POC PENK levels are associated with poorer 30-day, 90-day, and 1-year survival, as well as with a higher risk of developing in-hospital renal deterioration. Although similar associations have been previously described [15,16,17,18], it is important that such observations can be reproduced with the use of POC testing in the ED, where prompt stratification and management of patients with AHF is of utmost importance. Previous studies have utilized assays with various turnaround times and questionable clinical applicability [7,13], especially in the acute care setting.

Our AHF patient population displayed median PENK levels of 111 [60–193] pmol/L, being slightly higher than previously reported levels in AHF studies, which ranged from 86.2 to 104.9 pmol/L [15,17,18,19,20]. This discrepancy may be attributed to several factors. First, there are differences in the enrolment criteria between our study and the other AHF trials, as our eligibility criteria allowed the recruitment of patients, regardless of their AHF phenotype. As a result, patients presenting to the ED with cardiogenic shock qualified for the present study, since it followed an all-comer design. This patient subgroup represents the most severe form of AHF, carries the worst prognosis, and has been shown to exhibit very high PENK levels. Indeed, patients with cardiogenic shock from the CARDSHOCK trial have been reported to demonstrate high baseline median PENK levels of 105 pmol/L [19]. Another significant difference is that our study was performed after the incorporation of angiotensin receptor–neprilysin inhibitors (ARNIs) in the guideline-directed medical therapy for HF with a reduced ejection fraction [10], and, thus, a number of patients were already receiving ARNIs. Enkephalins are known substrates of neprilysin, and neprilysin inhibition has been shown to increase the levels of PENK. Although the observed positive association between the use of ARNIs and the elevation of PENK concentrations in plasma was lost at the 2-year follow-up [21], it would be reasonable to assume that patients who presented within 2 years of ARNI initiation may have had elevated baseline PENK levels. In addition, the timing of sample acquisition may also account for the higher median PENK levels in our study compared with previous ones. Contrary to other studies in which the blood sample may have been acquired several hours post-admission [15], all samples in our center were drawn immediately upon presentation, before administering any treatment. Inter-assay variabilities may have also influenced PENK measurements, although the POC assay demonstrated a strong correlation with the reference method during its analytical performance [12].

In our study, POC PENK was associated with AHF severity, as evidenced by its positive correlation with worse clinical and laboratory indices upon ED presentation. Higher levels of circulating PENK were observed in patients who were more critically ill, namely, those who presented with cardiogenic shock, displayed poorer hemodynamic and metabolic profiles, had higher levels of myocardial injury (troponin) and stretch (NT-proBNP) biomarkers, or required invasive mechanical ventilation. Indeed, the increase in enkephalin synthesis in the AHF setting is considered a counter-response to the overt neurohormonal activation that prevails in AHF and is intended to mitigate the underlying adrenergic hyperactivity by exerting opposing cardiorenal effects, namely, cardiodepression and diuresis [5].

Furthermore, we demonstrated that POC PENK is an independent predictor of all-cause mortality at different time points, after adjusting for potential confounders, such as age, eGFR, and NT-proBNP. Higher levels are associated with poor survival, which can be attributed to the fact that patients in the third tertile of POC PENK presented with more severe AHF. From a pathophysiological standpoint, the pronounced enkephalinergic response, reflected by elevated PENK levels, may lead to a strong cardiodepressive effect and aggravate HF [5]. Indeed, opioid antagonism has been shown to improve hemodynamics in patients with advanced AHF and elevated circulating enkephalins [6]. At an optimal cut-off value of 95.8 pmol/L, POC PENK displayed an AUC of >0.7 for predicting mortality at any time point, be it 30 days, 90 days, and 1 year post-admission. This finding is in line with evidence from previous studies regarding the prognostic role of PENK in terms of mortality [15,17,18] and suggests that POC PENK may assist in risk stratifying patients not only during the critical phase of decompensation, which encompasses the hospitalization period spanning from ED admission to hospital discharge, but also in the post-discharge period, which represents a crucial vulnerable phase over the course of HF management [10]. Yet, further research is warranted in order to confirm PENK’s unequivocal clinical utility by directly comparing the prognostic accuracy of PENK with that of other biomarkers. Ng et al. reported that the addition of both NT-proBNP and PENK to risk stratification clinical scores results in a higher c-statistic for predicting mortality at 1 year than the addition of either biomarker alone [15]. Such an observation supports the rationale that PENK should be incorporated into well-established scoring systems of patient risk classification rather than replace routine prognostic biomarkers, such as natriuretic peptides.

Moreover, PENK was associated with worse baseline renal function, demonstrating positive correlations with serum urea and creatinine. More importantly, PENK levels were able to independently predict WRF after adjustment for multiple other predictors of renal deterioration, yielding an AUC of 0.690 at an optimal cut-off of 90.5 pmol/L. Contrary to routine biomarkers that have been criticized for their role in predicting renal deterioration due to slow and late elevations in their levels, resulting in diagnostic delays, PENK appears to reflect real-time changes in renal function [9]. Owing to its small molecular weight, PENK has been studied as a novel glomerular filtration marker in both AHF and non-HF populations and has demonstrated promising results in predicting renal deterioration in a timely manner [8,9]. In particular, following acute kidney injury in patients with AHF, PENK levels increase presumably due to perturbations in glomerular filtration and diminished clearance, coupled with an increase in the synthesis of biologically active enkephalins in response to HF decompensation [5]. However, in patients with a history of ESRD undergoing hemodialysis, the association between PENK and renal and cardiac status is lost due to ‘falsely’ elevated levels of PENK [22]. This paradoxical increase following hemodialysis presumably occurs due to the disproportionate removal of fluid, which exceeds the elimination of PENK, and was the rationale for excluding this subgroup from our study. Nonetheless, in patients with AHF who develop WRF, PENK levels display an earlier increase than the standard-of-care creatinine, as evidenced by differences in the temporal pattern of kinetics of PENK and creatinine, wherein baseline PENK levels are elevated immediately upon ED presentation, whereas there is a delayed and disproportional rise in creatinine levels in patients who subsequently develop WRF [15]. Albeit modest, the predictive accuracy of POC PENK for WRF in our study (AUC: 0.690) is in accordance with previous large-scale studies conducted in AHF patients, which have reported similar AUCs of 0.652 [17] for short-term WRF, and 0.643 [18] for long-term WRF. Similar values have been recorded in patients with cardiogenic shock [19], whereas only one small-sized study has reported a much superior predictive performance of PENK for WRF [16]. Even though not extensively studied, there is evidence that PENK outperforms other glomerular and tubular injury markers in predicting WRF [18]. Therefore, POC PENK may provide early and valuable insights into renal function in AHF patients immediately upon ED presentation. Providing such information in a timely manner is particularly important, since it could minimize the number of patients with renal injury who initially go undetected and aid in tailoring the appropriate treatment. However, it should be noted that conflicting evidence also exists, since one study concluded that PENK exhibits limited prognostic value on top of established renal markers [20].

### Strengths and Limitations

To the best of our knowledge, this is the first study to assess the prognostic value of POC PENK in terms of predicting incident kidney injury and short- and long-term mortality in patients with AHF in the ED. Although similar results regarding its prognostic role have been previously described [15,17,19,20], PENK measurement in these studies was cumbersome, since it required sample centrifugation, freezing, and transport to specialized laboratories. On the contrary, our study has shown that the measurement of PENK by means of a POC assay is feasible in the ED setting and requires a short turnaround time. Besides that, an important finding of our study is that POC PENK predicts both mortality and WRF in patients with AHF upon ED presentation. Regarding its limitations, our study was carried out in a single center and included a rather small sample size, which may influence the strength of the results. Moreover, the sample size was not calculated prospectively, and a post hoc analysis was conducted to assess the power of the study based on the observed data. The enrolled population included only hospitalized patients with AHF, and, therefore, our findings cannot be extrapolated to individuals treated in outpatient settings. Owing to the observational nature of this study, only the clinical utility of PENK as a prognostic biomarker has been demonstrated, while no conclusions can be drawn about any existing causality or underlying pathophysiological mechanism.

## 5. Conclusions

POC PENK measured upon ED presentation independently predicts WRF, as well as short and long-term mortality in patients with AHF. Given the strong association of POC PENK with critical short-term outcomes, in particular, 30-day mortality and WRF, it is plausible that measurement of POC PENK may facilitate risk stratification, inform clinical decision making in the ED, and optimize patient management in an effective and timely manner. Further research is needed to assess whether POC PENK provides additional prognostic value for adverse outcomes, not captured by current biomarkers or clinical scores.

## Figures and Tables

**Table 1:** Baseline characteristics of study patients categorized by PENK tertiles.

	All Patients (n = 107)	PENK Tertile 1 (<50–76.4) (n = 36)	PENK Tertile 2 (76.5–166.5) (n = 36)	PENK Tertile 3 (166.6 - >500) (n = 35)	*p*-Value
**Demographics**					
Age (years), mean (SD)	72 (13)	68 (12)	71 (15)	78 (9)	0.003
Men, n (%)	62 (58%)	23 (64%)	21 (58%)	18 (51%)	0.567
**AHF phenotype, n (%)**					<0.001
Pulmonary edema	26 (24%)	8 (22%)	11 (30%)	7 (20%)	
Acutely decompensated chronic HF	66 (62%)	27 (75%)	19 (53%)	20 (57%)	
Cardiogenic shock	10 (9%)	1 (3%)	1 (3%)	8 (23%)	
Right HF	5 (5%)	0	5 (14%)	0	
**Comorbidities, n (%)**					
Arterial hypertension	52 (49%)	14 (39%)	23 (64%)	15 (44%)	0.082
Atrial fibrillation	47 (44%)	16 (44%)	17 (47%)	14 (41%)	0.878
Coronary artery disease	37 (35%)	15 (42%)	9 (25%)	13 (38%)	0.295
Prior MI	27 (25%)	10 (28%)	7 (19%)	10 (29%)	0.586
Permanent pacemaker	12 (11%)	2 (6%)	6 (17%)	4 (12%)	0.329
COPD	23 (22%)	8 (22%)	8 (23%)	7 (21%)	0.973
Thyroid disease	26 (24%)	6 (17%)	9 (25%)	11 (32%)	0.312
Diabetes mellitus	50 (47%)	20 (56%)	14 (39%)	16 (47%)	0.367
Chronic kidney disease	15 (14%)	3 (8%)	3 (8%)	9 (27%)	0.044
Stroke	12 (11%)	4 (11%)	5 (14%)	3 (9%)	0.799
Dyslipidemia	36 (34%)	12 (33%)	16 (44%)	8 (24%)	0.181
**Prior medications, n (%)**					
ACEi	16 (15%)	5 (14%)	4 (11%)	7 (21%)	0.497
ARB	27 (25%)	12 (34%)	6 (17%)	9 (27%)	0.233
ARNI	11 (10%)	2 (6%)	5 (14%)	4 (12%)	0.503
Beta-blockers	61 (57%)	20 (57%)	17 (47%)	24 (73%)	0.097
MRA	33 (31%)	7 (20%)	13 (36%)	13 (39%)	0.179
SGLT2i	11 (10%)	6 (17%)	3 (9%)	2 (7%)	0.327
Statins	47 (44%)	17 (49%)	15 (43%)	15 (46%)	0.891
Any diuretic	61 (57%)	17 (49%)	19 (53%)	25 (76%)	0.051
Antiplatelets	34 (32%)	11 (31%)	13 (36%)	10 (30%)	0.860
Anticoagulants	51 (48%)	17 (49%)	17 (47%)	17 (52%)	0.936
**ECG on admission, n (%)**					0.422
Sinus rhythm	58 (54%)	20 (56%)	20 (56%)	18 (51%)	
Atrial fibrillation/flutter	40 (37%)	15 (42%)	11 (31%)	14 (40%)	
Paced rhythm	8 (8%)	1 (3%)	5 (14%)	2 (6%)	
Ventricular tachycardia	1 (1%)	0	0	1 (3%)	
**Vital signs on admission**					
Heart rate (bpm), mean (SD)	100 (30)	104 (27)	95 (29)	100 (34)	0.407
SBP (mmHg), mean (SD)	137 (40)	144 (35)	144 (41)	122 (40)	0.037
DBP (mmHg), mean (SD)	77 (20)	84 (18)	78 (19)	70 (20)	0.012
**Focused echocardiography on admission**					
LVEF (%), median [IQR]	37 [25–50]	30 [26–40]	40 [21–50]	40 [25–50]	0.683
LVEF categories, n (%)					0.564
≤40	52 (49)	22 (61)	14 (42)	16 (49)	
41–49	21 (20)	6 (17)	7 (21)	8 (24)	
>50	29 (27)	8 (22)	12 (36)	9 (27)	
PASP (mmHg), median [IQR]	45 [30–55]	40 [30–50]	45 [33–55]	40 [30–55]	0.825
Right-ventricular diameter (mm), median [IQR]	34 [31–40]	33 [32–41]	34 [31–39]	35 [32–40]	0.736
IVC diameter					0.342
<15 mm, n (%)	7 (7%)	0	4 (13%)	3 (11%)	
15–25 mm, n (%)	44 (41%)	17 (53%)	13 (43%)	14 (50%)	
>25 mm, n (%)	39 (36%)	15 (47%)	13 (43%)	11 (39%)	
**Laboratory tests,** **median [IQR]**					
White blood cells, ×10^3^/μL	10.2 [7.9–13.0]	10.3 [8.4–12.4]	9.7 [6.6–13.4]	11.4 [8.3–13.1]	0.338
Hemoglobin, g/dL	12.5 [10.4–13.8]	12.8 [10.3–14.8]	12.8 [11.4–13.8]	11.7 [10.0–13.7]	0.180
Platelets, ×10^3^/μL	257 [202–315]	269 [210–320]	250 [210–293]	244 [199–353]	0.666
BUN, mg/dL	60 [38–96]	41 [32–58]	50 [37–70]	109 [71–155]	<0.001
Glucose, mg/dL	134 [103–185]	143 [96–198]	112 [97–151]	164 [125–219]	0.001
Serum creatinine, mg/dL	1.1 [0.9–1.7]	1.0 [0.8–1.2]	1.1 [0.8–1.5]	1.9 [1.5–2.4]	<0.001
eGFR, ml/min/1.73m^2^	62 [37–87]	81 [65–98]	68 [49–87]	32 [24–45]	<0.001
Serum sodium, mEq/L	137 [133–139]	138 [135–141]	139 [135–140]	134 [128–137]	0.001
Serum potassium, mEq/L	4.6 [4.1–5.2]	4.5 [4.1–5.0]	4.5 [4.0–4.9]	5.2 [4.3–6.1]	0.006
AST, U/L	24 [19–50]	22 [18–33]	25 [17–41]	31 [22–74]	0.050
ALT, U/L	19 [13–37]	19 [13–29]	17 [9–34]	20 [16–60]	0.127
CRP, mg/dL	15 [8–39]	10.1 [3.7–28.4]	21.8 [8.8–33.6]	28.6 [8.7–104.0]	0.081
hs-cTnT, pg/mL	39 [23–85]	29 [17–59]	30 [22–83]	71 [39–142]	<0.001
NT-proBNP, pg/mL	4684 [2607–12,049]	2932 [1992–4507]	6070 [2965–11,552]	9235 [3382–26,104]	0.001
PENK, pmol/L	111 [60–193]	NA	NA	NA	
**Blood gas analysis, median [IQR]**					
pH	7.41 [7.33–7.45]	7.42 [7.39–7.45]	7.42 [7.34–7.47]	7.38 [7.29–7.43]	0.031
pCO2, mmHg	39 [32–45]	38 [32–44]	38 [32–46]	40 [33–46]	0.829
HCO3, mEq/L	25 [21–28]	25 [22–29]	25 [21–28]	23 [19–29]	0.564
Lactate, mmol/L	1.7 [1.3–2.7]	1.6 [1.3–2.1]	1.5 [1.1–2.7]	2.0 [1.3–4.8]	0.251
Hypoxemia, n (%)	70 (65%)	27 (82%)	20 (65%)	23 (68%)	0.258
**Oxygen therapy at ED,** **n (%)**					
NIMV	19 (18%)	6 (17%)	6 (17%)	7 (20%)	0.914
IMV	3 (3%)	0	0	3 (9%)	0.042
**Length of stay (days),** **median [IQR]**	7 [5–11]	7 [5–9]	7 [6–12]	8 [5–15]	0.162

ACEi: angiotensin-converting enzyme inhibitor; AHF: acute heart failure; ARB: angiotensin II receptor blocker; ARNI: angiotensin receptor–neprilysin inhibitor; ALT: alanine aminotransferase; AST: aspartate aminotransferase; BUN: blood urea nitrogen; COPD: chronic obstructive pulmonary disease; CRP: C-reactive protein; DBP: diastolic blood pressure; ECG: electrocardiogram; ED: emergency department; eGFR: estimated glomerular filtration rate; HCO3: bicarbonate; HF: heart failure; hs-cTnT: high-sensitivity cardiac troponin T; IMV: invasive mechanical ventilation; IQR: interquartile range; IVC: inferior vena cava; LVEF: left ventricular ejection fraction; MI: myocardial infarction; MRA: mineralocorticoid receptor antagonist; NA: not applicable; NIMV: non-invasive mechanical ventilation; NT-proBNP: N-terminal pro B-type natriuretic peptide; PASP: pulmonary artery systolic pressure; pCO2: partial pressure of carbon dioxide; PENK: proenkephalin 119–159; SBP: systolic blood pressure; SD: standard deviation; SGLT2i: sodium–glucose cotransporter-2 inhibitor.

## Data Availability

The data are available from the corresponding author upon reasonable request.
